# Improved SARS-CoV-2 main protease high-throughput screening assay using a 5-carboxyfluorescein substrate

**DOI:** 10.1016/j.jbc.2022.101739

**Published:** 2022-02-17

**Authors:** Scott Legare, Fabian Heide, Ben A. Bailey-Elkin, Jörg Stetefeld

**Affiliations:** Department of Chemistry, University of Manitoba, Winnipeg, Manitoba, Canada

**Keywords:** high-throughput screening, FRET, viral protease, protease substrate, SARS-CoV-2, main protease (M^pro^), 3CL protease (3CL^pro^), enzyme kinetics, coronavirus, AMC, 7-amino-4-methylcoumarin, COVID-19, coronavirus disease 2019, DABCYL, 4-(4-dimethylaminophenylazo)benzoic acid, DLS, dynamic light scattering, DMSO, dimethyl sulfoxide, DNP, 2,4-dinitrophenyl, EDANS, 5-(2-aminoethylamino)-1-naphthalenesulfonic acid, FAM, 5-carboxyfluorescein, HCoV, human coronavirus, HTS, high-throughput screen, MCA, 7-methoxycoumarin-4-acetic acid, MERS-CoV, Middle East respiratory syndrome coronavirus, Mpro, main protease, nanoDSF, nano-differential scanning fluorimetry, PDB, Protein Data Bank, PLpro, papain-like protease, pp1a, polyprotein 1a, pp1ab, polyprotein 1ab, SARS-CoV-2, severe acute respiratory syndrome coronavirus 2

## Abstract

The emergence of severe acute respiratory syndrome coronavirus 2 (SARS-CoV-2) as a global threat to human health has highlighted the need for the development of novel therapies targeting current and emerging coronaviruses with pandemic potential. The coronavirus main protease (M^pro^, also called 3CL^pro^) is a validated drug target against coronaviruses and has been heavily studied since the emergence of SARS-CoV-2 in late 2019. Here, we report the biophysical and enzymatic characterization of native M^pro^, then characterize the steady-state kinetics of several commonly used FRET substrates, fluorogenic substrates, and six of the 11 reported SARS-CoV-2 polyprotein cleavage sequences. We then assessed the suitability of these substrates for high-throughput screening. Guided by our assessment of these substrates, we developed an improved 5-carboxyfluorescein-based FRET substrate, which is better suited for high-throughput screening and is less susceptible to interference and false positives than existing substrates. This study provides a useful framework for the design of coronavirus M^pro^ enzyme assays to facilitate the discovery and development of therapies targeting M^pro^.

Coronaviruses are a family of viruses commonly found in wildlife, companion animals, livestock, and humans. Human coronaviruses include HCoV-229E, HCoV-OC43, HCoV-NL63, and HCoV-HKU1 continuously circulating in the population and mostly cause mild symptoms associated with the common cold. In contrast, severe acute respiratory syndrome coronavirus (SARS-CoV), Middle East respiratory syndrome coronavirus (MERS-CoV), and SARS-CoV-2 are highly pathogenic coronaviruses causing the SARS epidemic, MERS epidemic, and most recently, the coronavirus disease 2019 (COVID-19) pandemic (1). To date, there have been over 220 million reported cases of COVID-19 and 4.6 million reported deaths. Despite the development of efficacious vaccines against COVID-19, SARS-CoV-2 transmission continues at high levels, and case numbers continue to increase (2, 3). As a result, there is an urgent need for effective antiviral drugs targeting SARS-CoV-2 that can be used to treat COVID-19.

SARS-CoV-2 is an enveloped positive-stranded RNA virus, which infects cells of the upper and lower respiratory tract. Upon entry into the host cell, the viral RNA genome is translated into two polyproteins, pp1a and pp1ab. These polyproteins are proteolytically processed by two viral proteases, a papain-like protease (PL^pro^) and a chymotrypsin-like main protease (M^pro^, also called 3CL^pro^), releasing 16 nonstructural proteins (1). M^pro^ inhibitors can effectively block SARS-CoV-2 replication in cell culture, demonstrating that M^pro^ is a valid drug target (4, 5, 6, 7, 8). SARS-CoV-2 M^pro^ has been shown to preferentially recognize the A-X-L-Q-(A/S) consensus sequence (where X is any amino acid), with cleavage occurring after the glutamine (9, 10). Interestingly, other coronaviruses including the related SARS-CoV and MERS-CoV share similar substrate specificity with SARS-CoV-2 M^pro^, suggesting that inhibitors of SARS-CoV-2 M^pro^ could serve as broad-spectrum antiviral drugs against future epidemic- or pandemic-causing coronaviruses (4, 11).

Discovery of M^pro^ inhibitors has relied heavily on the use of high-throughput screens (HTS) using a FRET-based peptide substrate to monitor protease activity (5, 7, 12, 13, 14, 15, 16, 17). Fluorogenic substrates that cleave an aminocoumarin-based fluorophore attached to the carboxyl terminus of a peptide have also been used (14, 15, 18). A number of M^pro^ enzyme assays have been developed using different substrates, M^pro^ constructs, and buffer conditions (14, 15, 19, 20, 21, 22). As a result, there have been inconsistent findings regarding the identification of potential M^pro^ inhibitors (20, 21, 23). This has highlighted a clear need for an improved SARS-CoV-2 M^pro^ assay that delivers better performance and improved consistency.

Here, we provide the detailed biophysical and enzymatic characterization of SARS-CoV-2 M^pro^ with native N termini and C termini and assess the steady-state kinetic parameters of three commonly used SARS-CoV-2 M^pro^ fluorescent substrates (4, 7, 18). We measured the kinetic efficiency of six SARS-CoV2 M^pro^ polyprotein cleavage sequences to determine the optimal substrate amino acid sequence. Guided by these results, an improved 5-carboxyfluorescein (FAM)-based FRET substrate was developed that is better suited for HTS and is less susceptible to interference and false positives than previously reported substrates. This study provides a useful framework for the design of assays aimed at discovering and developing new coronavirus M^pro^ inhibitors.

## Results

**Figure 1:**
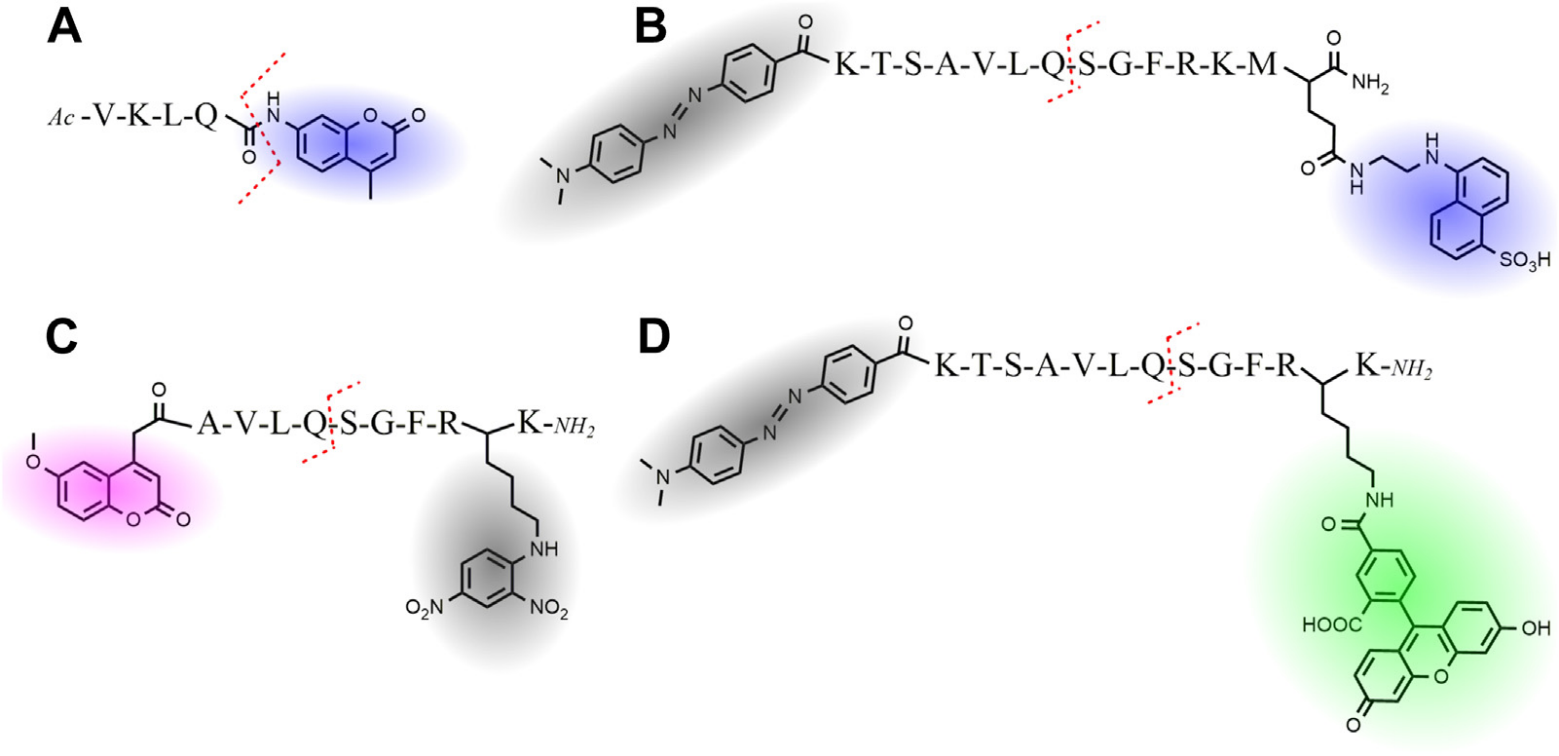
**Structure and names of fluorescent substrates used in this study.***A*, fluorogenic VKLQ–AMC substrate, with AMC fluorophore shown in *blue*. *B*, nsp4–5-EDANS FRET substrate uses an EDANS fluorophore shown in *blue*, and a 4-(4-dimethylaminophenylazo)benzoic acid (DABCYL) quencher shown in *gray*. *C*, nsp4–5-MCA FRET substrate uses an MCA fluorophore shown in *purple* with a 2,4-dinitrophenyl (DNP) quencher shown in *gray*. *D*, nsp4–5-FAM FRET substrate uses a FAM fluorophore shown in *green* with a DABCYL quencher shown in *gray*. The *red dashed line* indicates the cleavage site on the substrates. AMC, 7-amino-4-methylcoumarin; EDANS, 5-(2-aminoethylamino)-1-naphthalenesulfonic acid; FAM, 5-carboxyfluorescein; MCA, 7-methoxycoumarin-4-acetic acid.

Both fluorogenic and FRET-based substrates were used in this work (Fig. 1). The previously reported VKLQ–7-amino-4-methylcoumarin (AMC) fluorogenic substrate consists of a short peptide with a cleavable AMC fluorophore at the P1′ position (Fig. 1*A*) (18). The FRET substrates consist of a fluorophore and quencher pair separated by a SARS-CoV-2 polyprotein cleavage sequence. The previously reported FRET nsp4–5-5-(2-aminoethylamino)-1-naphthalenesulfonic acid (EDANS) substrate uses an EDANS fluorophore and a 4-(4-dimethylaminophenylazo)benzoic acid quencher (Fig. 1*B*), whereas the nsp4–5-7-methoxycoumarin-4-acetic acid (MCA) substrate uses an MCA fluorophore with a 2,4-dinitrophenyl quencher (Fig. 1*C*) (4, 7). The FAM-based FRET substrate developed in this work uses a 4-(4-dimethylaminophenylazo)benzoic acid quencher (Fig. 1*D*). Table 1 lists the amino acid sequence, cleavage site location, fluorophore, and quencher used for each substrate tested in this work.

**Table 1:** Steady-state kinetic parameters for SARS-CoV-2 M^pro^ fluorescent substrates

Substrate	Sequence^a^	*k*_cat_/*K*_*m*_ (M^−1^ s^−1^)
AKLQ–AMC	Ac-VKLQ ↓ {AMC}	18.5 ± 1.0
		24.5 ± 5.0^b^
nsp4–5-MCA	{MCA}-AVLQ ↓ SGFR{K(DNP)}K-*NH*_*2*_	14,190 ± 420
nsp4–5-EDANS	{DABCYL}-KTSAVLQ ↓ SGFRKM{E(EDANS)}-*NH*_*2*_	1960 ± 190
nsp4–5-FAM	{DABCYL}-KTSAVLQ ↓ SGFR{K(FAM)}K-*NH*_*2*_	2448 ± 85
nsp5–6-FAM	{DABCYL}-KSGVTFQ ↓ SAVK{K(FAM)}K-*NH*_*2*_	77.5 ± 4.2
nsp6–7-FAM	{DABCYL}-KKVATVQ ↓ SKMS{K(FAM)}K-*NH*_*2*_	68 ± 10
nsp8–9-FAM	{DABCYL}-KSAVKLQ ↓ NNEL{K(FAM)}K-*NH*_*2*_	6.01 ± 0.61
nsp10–12-FAM	{DABCYL}-KREPMLQ ↓ SADA{K(FAM)}K-*NH*_*2*_	4.74 ± 0.48
nsp14–15-FAM	{DABCYL}-KTFTRLQ ↓ SLEN{K(FAM)}K-*NH*_*2*_	38.0 ± 5.2

a Text in brackets denotes fluorophore or quencher, ↓ denotes cleavage site.

b Kinetic parameters determined from Michaelis–Menten plot. Each value reported as the mean ± 1 standard deviation, n = 3.

### Biophysical characterization demonstrates excellent protein quality

**Figure 2:**
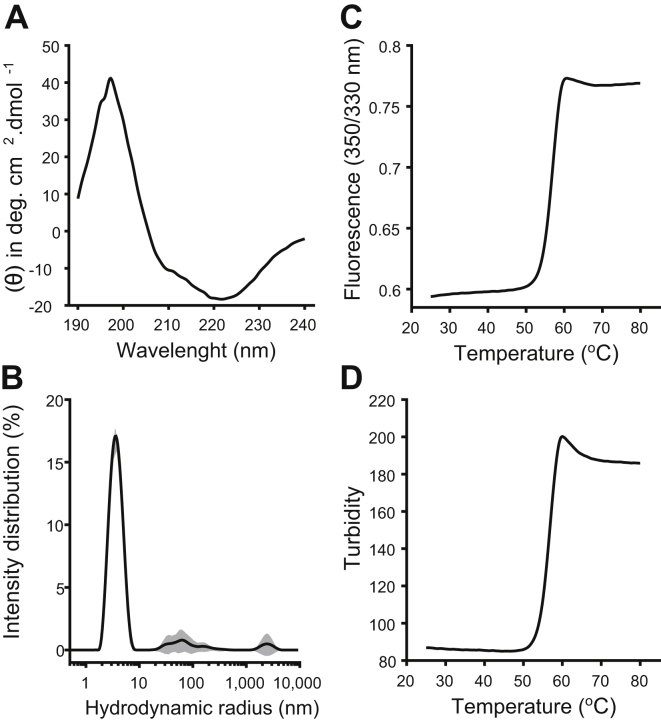
**Biophysical characterization of SARS-CoV-2 M**^**pro**^**.***A*, CD measurement showing secondary structure content of M^pro^. *B*, DLS intensity distribution with major peak showing a hydrodynamic radius (*R*_H_) of 3.76 ± 0.14 nm corresponding to M^pro^, *black line* shows mean intensity distribution with ±1 standard deviation shown in *gray-shaded area*, n = 10. *C*, 350/330 nm fluorescence ratio shows a melting onset at 51.1 °C and a *T*_m_ of 56.8 °C. *D*, turbidity shows an onset in aggregation beginning at 47.9 °C with an inflection point at 56.8 °C. DLS, dynamic light scattering; M^pro^, main protease; SARS-CoV-2, severe acute respiratory syndrome coronavirus 2.

Following the method developed by Xue *et al.* (24), SARS-CoV-2 M^pro^ with native N and C termini was expressed and purified to apparent homogeneity (Fig. S1) for further biophysical and enzymatic characterization. Nano-differential scanning fluorimetry (nanoDSF), dynamic light scattering (DLS), and CD spectroscopy were used to establish the quality of M^pro^ used in this work. CD measurements confirmed that the M^pro^ secondary structure is in agreement with what is expected based on the crystal structure (Protein Data Bank [PDB] ID: 6Y2E) (Fig. 2*A* and Table S1). DLS was used to measure the hydrodynamic radius (*R*_H_) of M^pro^ and to measure the state of M^pro^ self-oligomerization (Fig. 2*B*). Using the size distribution analysis model, the major intensity peak had an *R*_H_ of 3.76 ± 0.14 nm with a polydispersity index of 0.18 ± 0.03. The measured *R*_H_ of 3.76 ± 0.14 nm agrees with the expected *R*_H_ for the M^pro^ dimer based on the crystal structure (PDB ID: 6Y2E). The nanoDSF melt curve measured by the 350/330 nm fluorescence ratio showed a melting onset beginning at 51.1 °C and an inflection point or melting point (*T*_m_) of 56.8 °C (Fig. 2*C*). The baseline turbidity measurement is stable from 20 °C until the onset in turbidity increased beginning at 47.9 °C, indicating M^pro^ was stable against aggregation until 3.2 °C before the onset of melting begins (Fig. 2*D*). The inflection point of the turbidity measurement was 56.8 °C corresponding to the *T*_m_ of M^pro^. The measured *T*_m_ of 56.8 °C was slightly higher than previously reported values of between 48.5 and 54.2 °C (25, 26). Taken together, these results show M^pro^ is highly pure, properly folded, thermodynamically stable, and monodisperse in solution with very little aggregation or higher order oligomerization present.

### Optimization of assay buffer conditions

**Figure 3:**
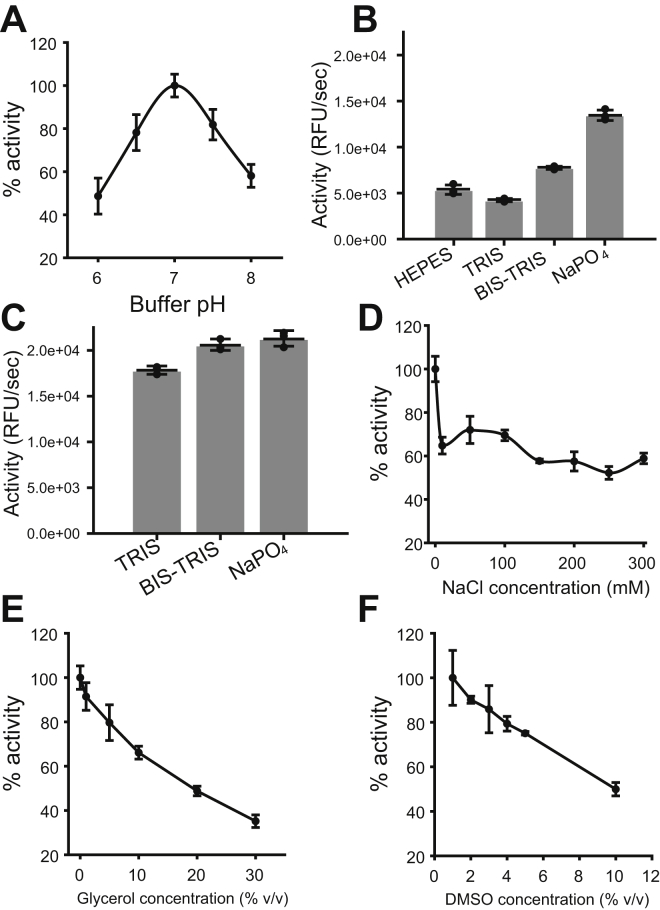
**Assessing the effects of common buffer conditions on M**^**pro**^**initial rate.** All tests are done in 100 μl buffer containing 100 μM VKLQ–AMC substrate and 200 nM enzyme. *A*, pH optimization in 20 mM NaPO_4_ (pH 6.0–8.0) with 150 mM NaCl. *B*, preference of M^pro^ for Hepes, Tris, Bis–Tris, or NaPO_4_, in 20 mM buffering agent, pH 7.0, with 150 mM NaCl. *C*, preference of M^pro^ for Tris, Bis–Tris, and NaPO_4_ in 20 mM buffering agent, pH 7.0. *D*, effect of 0 to 300 mM NaCl in 20 mM NaPO_4_ (pH 7.0). *E*, effect of 0 to 30% v/v glycerol in 20 mM NaPO_4_ (pH 7.0) with 150 mM NaCl. *F*, effect of 1 to 10% v/v DMSO in 20 mM NaPO_4_ (pH 7.0) with 150 mM NaCl. Each measurement is reported as the mean with error bars showing ±1 standard deviation, n = 3. AMC, 7-amino-4-methylcoumarin; DMSO, dimethyl sulfoxide; M^pro^, main protease.

The effects of different buffer conditions on M^pro^ activity were assessed to determine optimal assay conditions. The AKLQ–AMC substrate was chosen for buffer optimization because it displayed good solubility up to a concentration of around 2.5 mM in all buffers tested. The optimal pH for M^pro^ was found to be pH 7.0 (Fig. 3*A*). M^pro^ had a strong preference for NaPO_4_ as a buffering agent, followed by Bis–Tris, Hepes, and Tris, when tested at 20 mM buffering agent, 150 mM NaCl, pH 7.0 (Fig. 3*B*); however, this preference was lessened when tested at 20 mM buffering agent (pH 7.0) without NaCl (Fig. 3*C*). The highest enzyme activity was achieved when NaCl was omitted from the buffer while adding between 10 and 300 mM NaCl decreases the enzyme activity roughly the same degree (Fig. 3*D*). Both glycerol and dimethyl sulfoxide (DMSO) were found to have a negative effect on enzyme activity (Fig. 3, *E* and *F*). Based on these results, the optimized buffer found in this study consists of 20 mM NaPO_4_ at pH 7.0. It was found that the FRET substrates used in this work showed better solubility in buffers of low ionic strength; therefore, 20 mM Bis–Tris (pH 7.0) was used instead of a NaPO_4_-based buffer. These optimized buffer conditions closely agree with other work showing that M^pro^ is most thermodynamically stable at pH 7.0 in the absence of NaCl (26).

Measuring the reaction progress curve for the complete hydrolysis of substrate can help assess and identify nonoptimal buffer conditions and loss of enzyme activity because of inactivation and inhibition. It is also helpful to verify that the measured initial rate corresponds to the true linear steady-state portion of the reaction progress curve (27). When hydrolyzing the FRET substrates, the M^pro^ began to lose activity after about 400 s of reaction time in 20 mM Bis–Tris (pH 7.0) (Fig. S2). Adding 2 mM EDTA and 2 mM DTT to the assay buffer prevented this inactivation for the nsp4–5-FAM and nsp4–5-EDANS substrates (Fig. S2, *B* and *C*) but not for the nsp4–5-MCA substrate (Fig. S2*D*), which was inactive in 20 mM Bis–Tris (pH 7.0) and 2 mM EDTA. As previously discussed, the FRET substrates showed reduced solubility in buffers with higher ionic strength. The inactivity of the nsp4–5-MCA substrate in the 20 mM Bis–Tris (pH 7.0) and 2 mM EDTA buffer may be caused by the increase in ionic strength of the buffer from the addition of 2 mM EDTA, reducing the solubility of the nsp4–5-MCA substrate. In contrast to the FRET substrates, M^pro^ did not fully lose activity when hydrolyzing the VKLQ–AMC substrate (Fig. S2*A*), and addition of EDTA and DTT to the reaction buffer had a minimal effect on the enzyme’s behavior. The linear initial rate portion of the reaction progress curve could be measured for at least the first 600 s of reaction time with the VKLQ–AMC substrate and about 200 s for the FRET substrates. This initial rate was unaffected by the addition of EDTA and DTT (Fig. S2).

### Substrate steady-state kinetic parameters

**Figure 4:**
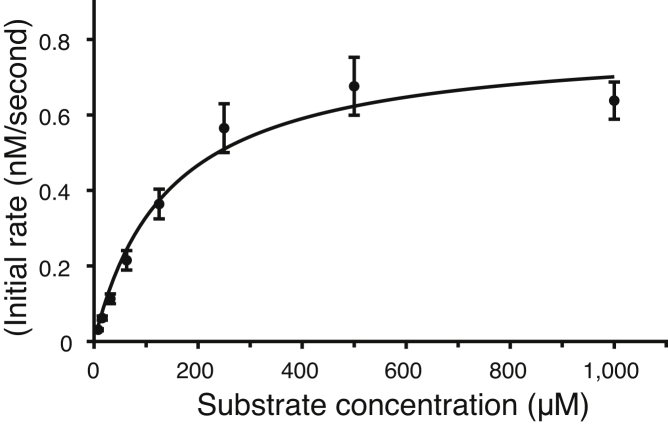
**Michaelis–Menten plot for the VKLQ–AMC substrate.** Values of 172 ± 28 μM, 0.842 ± 65 nM s^−1^; and 0.00421 ± 53 s^−1^ are measured for the *K*_*m*_, *V*_max_, and *k*_cat_, respectively. *k*_cat_/*K*_*m*_ was found to be 24.5 ± 5.0 M^−1^ s^−1^. Each data point is the mean with error bars showing ±1 standard deviation, n = 3. AMC, 7-amino-4-methylcoumarin.

Measurements were performed with each substrate to determine their utility for characterizing M^pro^. *k*_cat_/*K*_*m*_ was measured at low substrate concentrations where the initial rate increased linearly with substrate concentration. The results show that the nsp4–5-MCA substrate had the highest *k*_cat_/*K*_*m*_, followed by the nsp4–5-FAM and nsp4–5-EDANS substrates, whereas the VKLQ–AMC substrate had the lowest *k*_cat_/*K*_*m*_ value (Fig. S3, *A*–*D* and Table 1). The FRET substrates suffered from poor solubility and large inner filter effects when used at high concentrations needed to reach saturating substrate concentrations (*V*_max_). These are commonly reported problems for FRET substrates in general, including FRET substrates used for SARS-CoV-2 M^pro^ (18, 28, 29, 30). As a result, a full Michaelis–Menten plot that reaches saturating substrate concentrations could only be measured using the VKLQ–AMC substrate (Fig. 4 and Table 1).M^pro^ recognizes and cleaves 11 sites on the viral pp1a and pp1ab during viral replication. The nsp4–5 cleavage sequence at the N terminus of M^pro^ is the sequence commonly used in M^pro^ FRET substrates and is the sequence used in each of the nsp4–5-MCA, nsp4–5-EDANS, and nsp4–5-FAM substrates (Table 1). To test the kinetic efficiency of other SARS-CoV-2 polyprotein cleavage sequences, the *k*_cat_/*K*_*m*_ of five additional FAM-based FRET substrates utilizing the nsp5–6, nsp6–7, nsp8–9, nsp10–12, and nsp14–15 cleavage sequences were tested and compared with the nsp4–5-FAM substrate (Table 1). The *k*_cat_/*K*_*m*_ values for these substrates show that nsp4–5 has by far the highest *k*_cat_/*K*_*m*_ value followed by nsp5–6, nsp6–7, and nsp14–15, whereas nsp8–9 and nsp10–12 cleavage sites have by far the lowest *k*_cat_/*K*_*m*_ values (Fig. S3, *E*–*I* and Table 1). These results show that out of the substrates tested, the nsp4–5 sequence has the most desirable kinetic properties for use in enzyme assays.

### Characterizing the improved nsp4–5-FAM substrate

To assess the suitability of each of the three FRET substrates for HTS, the Z′-factor for each substrate was determined. The Z′-factor is a statistical parameter used to evaluate the quality of an HTS (31). The Z′-factor reflects two critical criteria that a good quality HTS must have. The first criterion is the signal dynamic range, which describes the difference in signal produced by a positive control and a negative control. When the assay signal dynamic range is large, the signal produced by an active compound can more confidently be distinguished from an inactive compound. The second criterion reflected in the Z′-factor is the variability or standard deviation of the signal produced by the positive and negative controls. When the positive and negative controls produce a consistent signal with low variability, the signal produced by an active compound can be more confidently differentiated from signal variability. To assess the Z′-factor for a FRET substrate, the mean and standard deviation of the initial rate measured for 16 positive and 16 negative controls was calculated. The signal dynamic range and Z′-factor were calculated as described in the Experimental procedures section. Baicalein is a noncovalent inhibitor of SARS-CoV-2 (32) and was used as a positive control; the negative control contained DMSO instead of baicalein. This assay was repeated in triplicate for each FRET substrate, with results reported in Table 2. Of the FRET substrates tested in this work, the nsp4–5-EDANS substrate produces the smallest standard deviation in signal for the positive and negative controls, followed by the nsp4–5-FAM and nsp4–5-MCA substrates. The nsp4–5-FAM substrate produced the largest signal dynamic range followed by the nsp4–5-MCA and nsp4–5-EDANS substrates (Table 2). The large signal dynamic range produced by the nsp4–5-FAM substrate is largely attributed to the much higher brightness of the FAM fluorophore in comparison to the MCA and EDANS fluorophores (Fig. S4). In this study, the nsp4–5-MCA and nsp4–5-EDANS substrates produced an Z′-factor of between 0.72 and 0.79, whereas the nsp4–5-FAM substrate produces a considerably higher Z′-factor of between 0.82 and 0.85 (Table 2).

**Table 2:** HTS assay quality statistics for SARS-CoV-2 M^pro^ FRET substrates

Substrate	Replicate	Signal mean (RFU/s)^a^		Signal standard deviation (RFU/s)^a^		Signal dynamic range (RFU/s)	Z′-factor
		Positive control	Negative control	Positive control	Negative control		
nsp4–5-EDANS	1	287	6182	141	315	5895	0.768
	2	699	6638	132	382	5939	0.741
	3	551	6022	179	297	5471	0.739
nsp4–5-MCA	1	1190	84,193	407	6521	83,003	0.750
	2	2389	98,870	646	6115	96,482	0.790
	3	3023	91,495	580	7568	88,472	0.724
nsp4–5-FAM	1	5004	162,872	575	8923	157,868	0.820
	2	3418	140,138	471	6135	136,720	0.855
	3	4194	156,189	1086	7013	151,955	0.840

Abbreviation: RFU, relative fluorescent unit.

a Signal mean and standard deviation, n = 16.

## Discussion

The SARS-CoV-2 M^pro^ is a validated drug target, and M^pro^ inhibitors have been shown to block viral replication in cell culture (4, 5, 6, 7, 8). In addition, M^pro^ inhibitors could have broad-spectrum antiviral activity against related coronaviruses because of the conserved features of the M^pro^ recognition sequence (4, 11). Fluorescent substrates are commonly used to study M^pro^ enzymatic activity, identify inhibitors through HTS, and test inhibitor efficacy. In this study, we perform a biophysical characterization of SARS-CoV-2 M^pro^ and assess the steady-state kinetic parameters of three commonly used substrates, as well as six polyprotein cleavage sequences. We then develop the improved nsp4–5-FAM substrate that is better suited for HTS when compared with commonly used FRET substrates, resulting from the higher brightness of the FAM fluorophore. In addition, the FAM fluorophore is less susceptible to interference and false positives because of the green-shifted absorption and emission spectra of the FAM fluorophore.

The protease used in this study was produced recombinantly in *Escherichia coli* following a previously described method (24). M^pro^ produced by this method has been successfully used for structural and enzymatic studies (4, 7, 8, 18). The primary advantage of this method is that it generates M^pro^ with native N and C termini, which are known to be structurally different, and more catalytically active than M^pro^ with non-native N or C termini (4, 24). In addition, conflicting M^pro^ enzymatic data have been published in the literature, which has been in part attributed to the use of different M^pro^ constructs with non-native termini (20, 21, 23). Work on the closely related SARS-CoV M^pro^ has recommended the standard adoption of native termini M^pro^ for enzymatic and structural studies (30). For these reasons, native SARS-CoV-2 M^pro^ was used for the biophysical and enzymatic work done in this study.

Characterization of substrate kinetic parameters is critical for understanding the behavior of both the substrate and enzyme and can also help guide the development of a properly optimized enzyme assay. *k*_cat_/*K*_*m*_ is an informative and useful parameter that gives a measure of substrate specificity and is the apparent second-order rate constant for product formation. We found that the value of 14,190 ± 420 M^−1^ s^−1^ for the nsp4–5-MCA substrate was six to seven times higher than the value of 2448 ± 85 M^−1^ s^−1^ and 1960 ± 190 M^−1^ s^−1^ measured for the nsp4–5-FAM and nsp4–5-EDANS substrates, respectively, which is consistent with values reported in the literature (4, 7). The *k*_cat_/*K*_*m*_ value of 18.5 ± 1.0 M^−1^ s^−1^ for the VKLQ–AMC substrate is far lower than the FRET substrates because of the shorter recognition sequence of the VKLQ–AMC substrate, which lacks the C-terminal residues to the cleavage site (18).

The VKLQ–AMC substrate was the only substrate that could be used at concentrations needed to reach *V*_max_. By measuring the Michaelis–Menten kinetics of the VKLQ–AMC substrate, we report a *k*_cat_/*K*_*m*_ of 24.5 ± 5.0 M^−1^ s^−1^, which agrees with the value of 18.5 ± 1.0 M^−1^ s^−1^ obtained using low substrate concentrations. This demonstrates that the behavior of the VKLQ–AMC substrate is consistent at low and high concentrations; therefore, the VKLQ–AMC substrate is relatively unaffected by the inner filter effect. Others have also found that these fluorogenic substrates are better suited for use at high concentrations than FRET substrates (18). A chromogenic substrate very similar to the fluorogenic VKLQ–AMC substrate was more useful for catalytic mechanism studies of SARS-CoV M^pro^ than the nsp4–5-EDANS FRET substrate (33).

Of the M^pro^ polyprotein cleavage sequences tested, we found that the nsp4–5 cleavage sequence has by far the highest *k*_cat_/*K*_*m*_ and therefore is best suited for use in enzyme assays. This result is consistent with measurements done on the SARS-CoV M^pro^, which also show that the nsp4–5 cleavage sequence has the highest *k*_cat_/*K*_*m*_ of the 11 polyprotein cleavage sequences (34). N-terminomics studies have identified the preferred cleavage sequence of SARS-CoV-2 M^pro^ to be A-X-L-Q↓(A/S) (9, 10). Of the cleavage sequences we tested, the nsp4–5 sequence is the only one that strictly represents this consensus sequence.

By characterizing the properties of the previously published nsp4–5-EDANS, nsp4–5-MCA, and VKLQ–AMC substrates, we recognized that an improved substrate for HTS could be developed. Because the *k*_cat_/*K*_*m*_ of the VKLQ–AMC substrate is low, high concentrations of substrate and enzyme are needed to generate a measurable fluorescent signal. This makes the VKLQ–AMC substrate undesirable for HTS because of the larger amounts of enzyme and substrate that would be consumed. We also recognized that both the nsp4–5-MCA and nsp4–5-EDANS substrates use fluorophores with relatively low brightness and undesirable excitation and emission spectra that makes them susceptible to interference from assay compounds (35). To develop an improved FRET substrate for HTS, we chose to use a FAM fluorophore because of its higher brightness and spectral properties that are less prone to interference. In addition, FAM is an inexpensive and readily available fluorophore that can easily be incorporated into the peptide substrate by most custom peptide synthesis companies.

To assess whether our new nsp4–5-FAM substrate is better for HTS than existing nsp4–5-MCA and nsp4–5-EDANS substrates, we characterized the Z′-factor for these substrates. An ideal assay produces an Z′-factor of 1; however, an experimental assay could never achieve this value. An Z′-factor of greater than 0.5 is usually considered an excellent quality assay (31). We found that both the nsp4–5-MCA and nsp4–5-EDANS substrates produced an Z′-factor of 0.75, indicating our assay conditions are well optimized. However, because of the low brightness of the MCA and EDANS fluorophores, these substrates produce a low-signal dynamic range, which limits the Z′-factor. The most effective way to develop a substrate that is better suited for HTS is to use a brighter fluorophore. We found that by using the brighter FAM fluorophore, we were able to greatly increase the signal dynamic range of the assay and increase the Z′-factor of the assay to 0.84. This demonstrates that the improved nsp4–5-FAM substrate is better suited for HTS.

False positives are another common issue encountered in HTS assays and are especially problematic when screening large libraries of compounds (35). Fluorescent-based assays are especially susceptible to false positives caused by compounds that interfere with the measured fluorescent signal. In addition, the vast majority of compounds tested in HTS absorb and fluoresce at wavelengths in the ultraviolet and blue regions of the spectrum (<490 nm) (36). This makes the nsp4–5-MCA and nsp4–5-EDANS substrates (excitation/emission of 320/405 nm and 350/480 nm, respectively) especially susceptible to interference and false positives. In contrast, the nsp4–5-FAM substrate absorbs and emits green light (excitation/emission of 490/530 nm) and is therefore largely unaffected by this source of interference, reducing the potential for false positives (35).

The biophysical and enzymatic characterization of the native SARS-CoV-2 M^pro^ described in this work will serve as a valuable reference for future studies investigating the activity of SARS-CoV-2 M^pro^. Using optimized assay conditions, we were able to compare properties of commonly used M^pro^ substrates and develop an improved nsp4–5-FAM substrate that is better suited for HTS. When compared with commonly used M^pro^ FRET substrates, this substrate generates a better-quality HTS assay because of the higher brightness of the FAM fluorophore and is less susceptible to interference from assay compounds because of its green-shifted absorbance and emission spectra. This substrate will thus serve as a valuable tool in the development and design of future HTS assays aimed at identifying and characterizing novel direct-acting antivirals targeting the SARS-CoV-2 M^pro^.

## Experimental procedures

### Construct design, enzyme expression, and storage

Design of the expression vector followed previously reported methods (4, 24). The codon-optimized SARS-CoV-2 M^pro^ open reading frame was inserted at the BamHI and XhoI restriction sites of a PGEX-6p-1 expression vector. The M^pro^ open reading frame contained the N-terminal autocleavage site AVLQ↓SGFRK (↓ denotes cleavage site) and a modified version of the C-terminal autocleavage site VTFQ↓GP followed by a His_6_ tag. Autocleavage occurs during protein expression to produce a native N terminus. The modified C-terminal autocleavage site is not cleaved by M^pro^ but can be cleaved by human rhinovirus 3C protease to produce the native M^pro^ C terminus during protein purification. This SARS-CoV-2 M^pro^ expression vector was synthesized by GenScript.

The SARS-CoV-2 M^pro^ expression vector was transformed into *E. coli* strain BL21-Gold (DE3) (37). A single colony was used to inoculate a 50 ml culture of Miller's LB containing 100 μg/ml ampicillin overnight at 30 °C with shaking. About 10 ml of overnight culture was used to inoculate 600 ml of LB containing 100 μg/ml ampicillin. This culture was grown at 37 °C until an absorbance of around 0.6 at 600 nm was reached and then induced with 0.5 mM IPTG for 14 h at 20 °C. Cells were harvested at 4 °C by centrifugation at 4000*g* for 20 min. The cell pellet was resuspended in a minimal volume of 20 mM Tris (pH 8.0), 300 mM NaCl, and stored at −20 °C. Cells were thawed and then lysed by sonication on ice. Lysate was clarified at 4 °C by centrifugation at 48,000*g* for 20 min. The supernatant was passed onto a 5 ml HisTrap HP (Cytiva) equilibrated with buffer A (20 mM Tris [pH 8.0], 20 mM imidazole, 500 mM NaCl, and 1 mM DTT). The column was washed with 25 ml of buffer A, then M^pro^ was eluted with a linear gradient from 0 to 100% buffer B (20 mM Tris [pH 8.0], 500 mM NaCl, 500 mM NaCl, and 1 mM DTT) over 75 ml, and fractions of 4 ml were collected. Fractions were analyzed by SDS-PAGE, those containing M^pro^ were pooled and mixed with human rhinovirus 3C protease (Sigma–Aldrich) in a 40:1 ratio, and dialyzed into 50 mM Tris (pH 8.0), 150 mM NaCl, 1 mM EDTA, and 1 mM DTT overnight at room temperature. Next, the protein was exchanged into buffer C (50 mM Tris [pH 8.0] and 1 mM DTT) and concentrated to 10 mg/ml using a 10 kDa nominal molecular weight centrifugal filter. The protein was loaded onto a Mono Q 4.6/100 PE anion exchange column (Cytiva) pre-equilibrated with buffer C. The column was washed with 20 ml of buffer C, then M^pro^ was eluted with a linear gradient from 0 to 30% buffer D (50 mM Tris [pH 8.0], 500 mM NaCl, and 1 mM DTT) over 20 ml, and 0.5 ml fractions were collected. Fractions were analyzed by SDS-PAGE, those containing pure M^pro^ free of detectable contamination were pooled and buffer exchanged into 20 mM Tris (pH 8.0), 150 mM NaCl, 1 mM Tris(2-carboxyethyl)phosphine, and concentrated using a centrifugal filter.

Protein concentration was measured spectrophotometrically using an extinction coefficient of 32,890 M^−1^ cm^−1^ and a molecular weight of 33,796 Da, calculated by ProtParam (38). M^pro^ was stored at a concentration of 45.3 μM in 50% v/v ethylene glycol at −80 °C for long-term storage and −20 °C for short-term storage. There was no substantial loss of enzyme activity under these storage conditions.

### nanoDSF and DLS

Thermodynamic stability and particle size distribution of M^pro^ were measured using a Prometheus Panta (NanoTemper Technologies GmbH). M^pro^ at a concentration of 1.1 mg/ml in 20 mM Tris (pH 8.0), 150 mM NaCl, 1 mM EDTA, 1 mM DTT was filtered through a 0.1 μm Ultrafree centrifugal filter (Merck Millipore). Standard Prometheus capillaries (PR-C002) were used. nanoDSF measurements were done from 25 to 80 °C with a temperature gradient of 1 °C/min, and intrinsic tryptophan fluorescence was measured at 350 and 330 nm. For DLS measurements, 10 acquisitions of 5000 ms each were done at a temperature of 20 °C with 100% DLS power. Buffer viscosity was calculated to be 1.02139 mPa•s using the buffer builder incorporated in the Panta Control software, version 1.2.1. The autocorrelation function was fit to a size distribution analysis model. Data analysis was done with the Panta analysis software, version 1.2.

### CD spectroscopy

CD measurements of M^pro^ were performed at a concentration of 0.5 mg/ml in 10 mM Na_2_HPO_4_ (pH 8.0). CD measurements were taken with a Jasco J-810 (JASCO Corporation) spectropolarimeter at 20 °C in a 0.05 cm path length quartz cuvette. Raw data were converted to mean residue ellipticity, and secondary structure deconvolution was done using the CDSSTR algorithm and the SMP180 reference set on the DichroWeb server (39, 40). Experimental secondary structure fractions were compared to the protease crystal structure (PDB ID: 6Y2E) using PDBsum (41).

### Fluorescent substrates

Amino acid sequences of the substrates used in this study can be found in Table 1 and Figure 1. The nsp4–5-MCA substrate was purchased from CanPeptide, Inc; all other substrates were purchased from GenScript. All substrates had a purity greater than 95% confirmed by HPLC and the molecular weight confirmed by mass spectrometry (testing done by supplier). The excitation and emission wavelengths used for each substrate are as follows: VKLQ–AMC 360/460 nm excitation/emission; nsp4–5-EDANS 350/480 nm excitation/emission; nsp4–5-MCA 320/405 nm excitation/emission; and FAM-based substrates 490/530 nm excitation/emission. All substrates came lyophilized as the trifluoroacetic acid salt; stock solutions were prepared in DMSO and stored protected from light at −20 °C.

### Enzyme assay general methods

All measurements were taken on a SpectraMax iD5 microplate reader controlled by Softmax pro 7.1 software (Molecular Devices). All readings were done in a black 96-well flat-bottom polypropylene microplate (Greiner Bio-One; ref 655209). Readings were taken every 20 s for 600 s to measure initial reaction rates and up to 1.5 h to measure complete hydrolysis. Measurements were done at ambient temperature. Initial rates were fit to the linear portion of the reaction progress, usually the first 200 s corresponding to less than 10% substrate hydrolysis. Fluorescence units were converted to concentration using a standard curve generated using a fluorophore standard in 20 mM Bis–Tris (pH 7.0). MCA and EDANS were purchased from Fisher Scientific; FAM was purchased from Cayman Chemical Company; and AMC was purchased from Sigma–Aldrich. All parameter fittings by linear and nonlinear regression were done in QtiPlot (IonDev Software). All measurements were performed in triplicate, and final values are expressed as the mean ± 1 standard deviation of the three measurements.

### Steady-state enzyme kinetics

*k*_cat_/*K*_*m*_ measurements were done in 20 mM Bis–Tris (pH 7.0) with a well volume of 100 μl. For the nsp4–5-FAM, nsp4–5-EDANS, and nsp4–5-MCA substrates, 80 nM enzyme was used with substrate ranging from 15 to 1.3 μM for nsp4–5-FAM and nsp4–5-EDANS or 15 to 0.88 μM for nsp4–5-MCA. For the VKLQ–AMC, nsp5–6-FAM, and nsp6–7-FAM substrates, 200 nM enzyme was used, with substrate concentrations ranging from 25 to 0.78 μM for the VKLQ–AMC substrate and 25 to 1.46 μM for the nsp5–6-FAM and nsp6–7-FAM substrates. For the nsp8–9-FAM and nsp14–15-FAM substrates, 200 nM enzyme was used with substrate concentrations from 50 to 2.93 μM. For the nsp14–15-FAM substrate, the baseline rate of substrate hydrolysis in the absence of enzyme was subtracted from the rate of hydrolysis with enzyme. For the nsp10–12-FAM substrate, 400 nM enzyme was used with substrate concentrations ranging from 100 to 13.2 μM. Initial rate measured in relative fluorescent unit/second was converted to M/second. This rate was divided by the molar concentration of enzyme used in the assay, to give the rate of product formation per second per enzyme active site in units of second^−1^. A plot of this rate against molar substrate concentration was made; the slope of this plot gives the value of *k*_cat_/*K*_*m*_. To measure the full Michaelis–Menten plot for the VKLQ–AMC substrate, 100 μl of 200 nM enzyme and between 1000 and 7.8 μM substrate in 20 mM Bis–Tris (pH 7.0) was used. Initial rate in relative fluorescent unit/second was converted to M/second. A plot of reaction rate in M/second *versus* the molar substrate concentration was fit to the Michaelis–Menten equation to obtain values of *K*_*m*_ and *V*_max_. To calculate *k*_cat_, *V*_max_ was divided by the molar concentration of enzyme used in the assay. With these values of *k*_cat_ and *K*_*m*_, the value of *k*_cat_/*K*_*m*_ can be calculated independently from the method described previously.

### HTS assessment

The Z′-factor was assessed by measuring enzyme activity of 16 positive and 16 negative controls and repeated in triplicate for each FRET substrate. Baicalein (CAS number: 491-67-8; Sigma–Aldrich), a noncovalent inhibitor of SARS-CoV-2 M^pro^, was used as a positive control; the negative control contained DMSO instead of baicalein. The reaction contained 100 μl of 10 μM substrate, 40 nM enzyme, and either 50 μM baicalein or DMSO as the positive and negative controls, respectively. The final buffer composition was 20 mM Bis–Tris (pH 7.0) and 1.2% v/v DSMO. For each assay, the mean and standard deviation of the initial rate for positive and negative controls were calculated. The signal dynamic range was calculated according to the following, where ${\bar{\mu }}_{n}$ and ${\bar{\mu }}_{p}$ are the mean of the negative and positive controls, respectively.

$$\text{Signal dynamic range}\;={\bar{\mu }}_{n}-{\bar{\mu }}_{p}$$

The Z′-factor was calculated according to Zhang *et al.* (31), where $\sigma_{p}$ and $\sigma_{n}$ are the standard deviation of the positive and negative controls, respectively.

$$Z^{\prime }=1-\;\frac{\left(3\sigma_{p}+3\sigma_{n}\right)}{\left|{\bar{\mu }}_{p}-{\bar{\mu }}_{n}\right|}$$

## Data availability

All data are contained within the article and in the supporting information.

## Supporting information

This article contains supporting information.

## Conflict of interest

The authors declare that they have no conflicts of interest with the contents of this article.
