# Supplementary material for: Improved SARS-CoV-2 main protease high-throughput screening assay using a 5-carboxyfluorescein substrate
Source: J Biol Chem. 2022 Feb 17;298(4):101739. doi: 10.1016/j.jbc.2022.101739 (PMC8849842; doi:10.1016/j.jbc.2022.101739)
Supplement: Supplemental Figures S1–S4 and Table S1 [file mmc1.pdf]

Supporting information

Improved SARS-CoV-2 main protease high throughput screening assay using a 5-carboxyfluorescein substrate

Scott Legare[1,]*, Fabian Heide[1], Ben A Bailey-Elkin[1], Jörg Stetefeld[1,]*

[1] Department of Chemistry, University of Manitoba, Winnipeg, Manitoba, Canada

*Corresponding authors:

Scott Legare - legares@myumanitoba.ca

Jörg Stetefeld - jorg.stetefeld@umanitoba.ca

Table of Contents

Figure S1. SDS-PAGE of SARS-CoV-2 M^{pro}.

Table S1. SARS-CoV-2 M^{pro} circular dichroism spectra analysis.

Figure S2. Reaction progress curve for the complete substrate hydrolysis.

Figure S3. Measurement of K_{cat}/K_m at substrate concentrations below substrate K_m.

Figure S4. Relative fluorescence units produced by fluorophores.

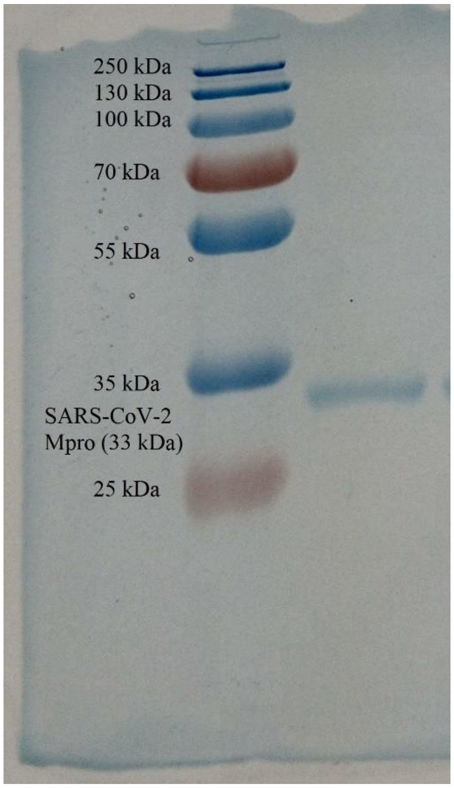

Figure S1. SDS-PAGE of SARS-CoV-2 M^{pro} used for biophysical and enzymatic studies.

Table S1. SARS-CoV-2 M^{pro} circular dichroism spectra analysis.

	Helix (%)	Sheet (%)	Turn (%)	Random coil (%)	Other[c] (%)
CDSSTR[a]	29	26	19	28	
PDB ID: 6Y2E[b]	25	27			48

[a] secondary structure content of CD spectra estimated by CDSSTR algorithm. [b] secondary structure of crystal structure estimated using PDBsum. [c] includes turns and random coils.

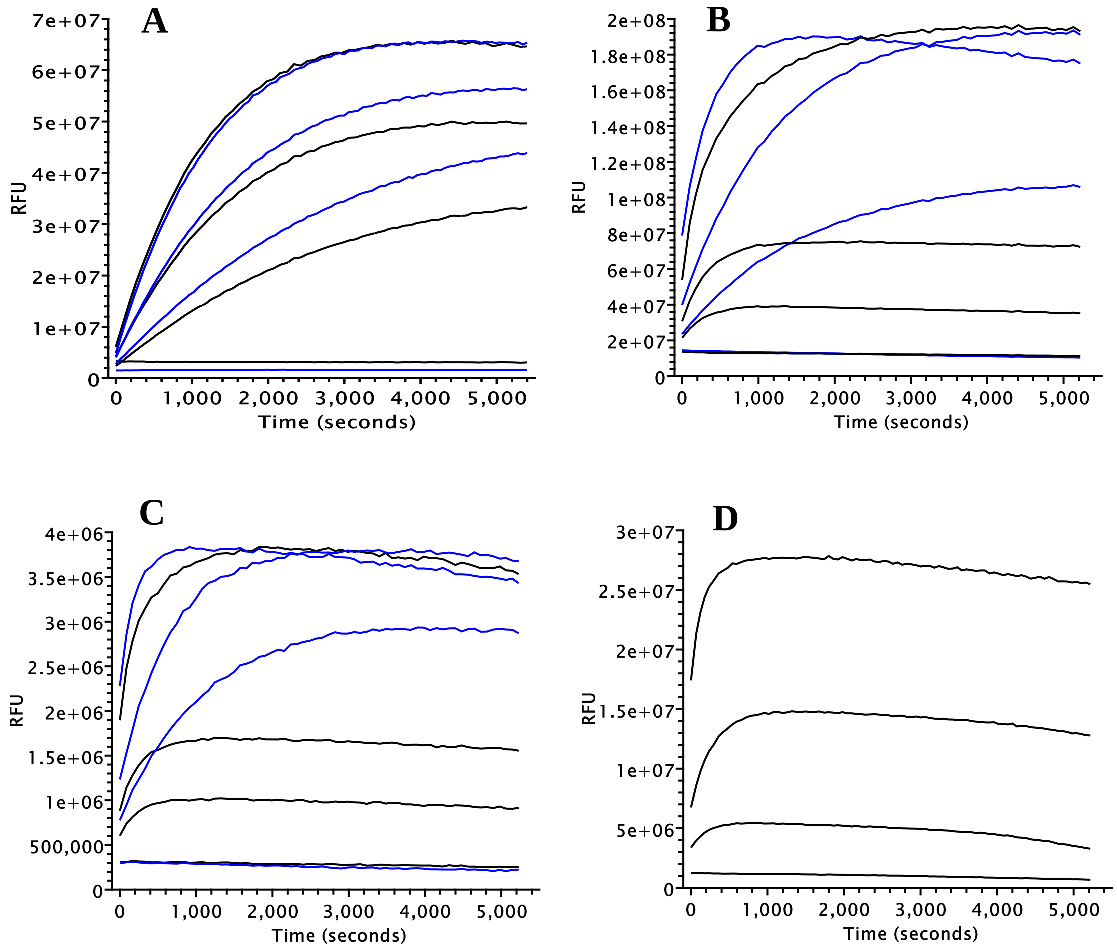

Figure S2. Reaction progress curve for the complete hydrolysis of substrate. 20 µM substrate in 20 mM BIS-TRIS pH 7.0 (black lines) and 20 mM BIS-TRIS pH 7.0, 2 mM EDTA, 2mM DTT (blue lines). Each condition was tested at four enzyme concentrations. *A*, For the VKLQ - AMC substrate 0, 2, 4 and 8 µM enzyme was used. *B-D*, For the nsp4-5-FAM, nsp4-5-EDANS, and nsp4-5-MCA substrates 0, 40, 80 and 160 nM enzyme was used. nsp4-5-MCA substrate was inactive in the presence of EDTA. Each curve represents the mean of 3 independent measurements.

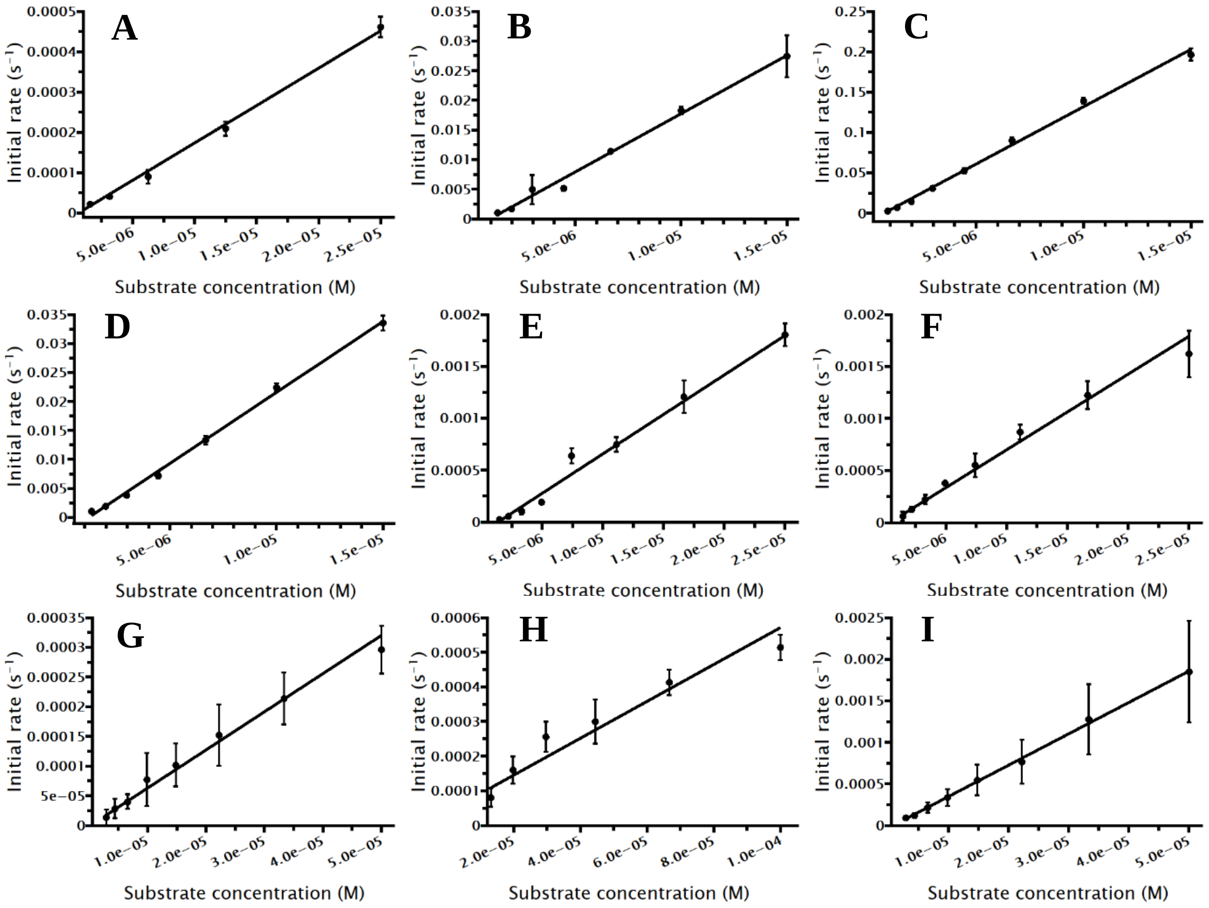

Figure S3. Measurement of K_{cat}/K_m at substrate concentrations below substrate K_m. Y axis shows the initial rate of product formation per second per enzyme active site in units of s^{-1}. *A*, VKLQ - AMC substrate. *B*, nsp4-5-EDANS substrate. *C*, nsp4-5-MCA substrate. *D*, nsp4-5-FAM substrate. *E*, nsp5-6-FAM substrate. *F*, nsp6-7-FAM substrate. *G*, nsp8-9-FAM substrate. *H*, nsp10-12-FAM substrate. *I*, nsp14-15-FAM substrate. Each data point is the mean ± 1 standard deviation, n = 3.

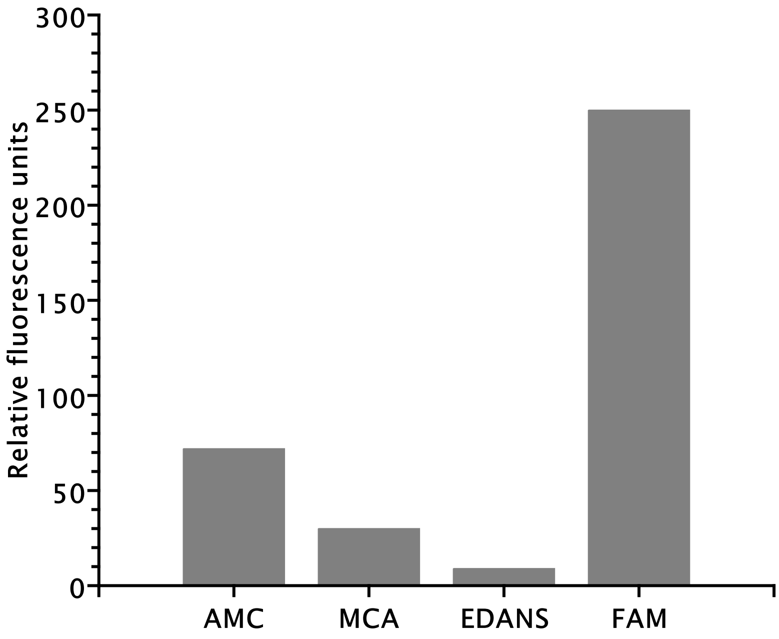

Figure S4. Relative fluorescence units produced by 100 µL of 2 µM fluorophore in 20mM BIS-TRIS pH 7.0. This shows the FAM fluorophore is much brighter in comparison to other fluorophores used in the fluorescent substrates. Values are reported as the mean of 3 measurements.
